# Supplementary material for: Cellular variability of nonsense-mediated mRNA decay
Source: Nat Commun. 2021 Dec 10;12:7203. doi: 10.1038/s41467-021-27423-0 (PMC8664836; doi:10.1038/s41467-021-27423-0)
Supplement: Supplementary file 1 — Supplementary Information [file 41467_2021_27423_MOESM1_ESM.pdf]

Supplementary Information

Title: Cellular variability of nonsense-mediated mRNA decay

Author list: Hanae Sato[1] and Robert H. Singer[1,2,3*]

Affiliations:

[1]Department of Anatomy and Structural Biology, Albert Einstein College of Medicine, 1300 Morris Park Ave, Bronx, NY 10461, USA

[2]Department of Cell Biology, Albert Einstein College of Medicine, 1300 Morris Park Ave, Bronx, NY 10461, USA

[3]Gruss Lipper Biophotonics Center, Albert Einstein College of Medicine, 1300 Morris Park Ave, Bronx, NY 10461, USA

*Correspondence should be addressed to:

Robert H. Singer, PhD

Phone: +1.718.430.8646

Fax: +1.718.430.8697

Email: robert.singer@einsteinmed.org

Office: Golding Building Room 601A

This PDF includes:

Supplementary Figures 1 to 9

Supplementary Table 1 to 2

Supplementary Figures

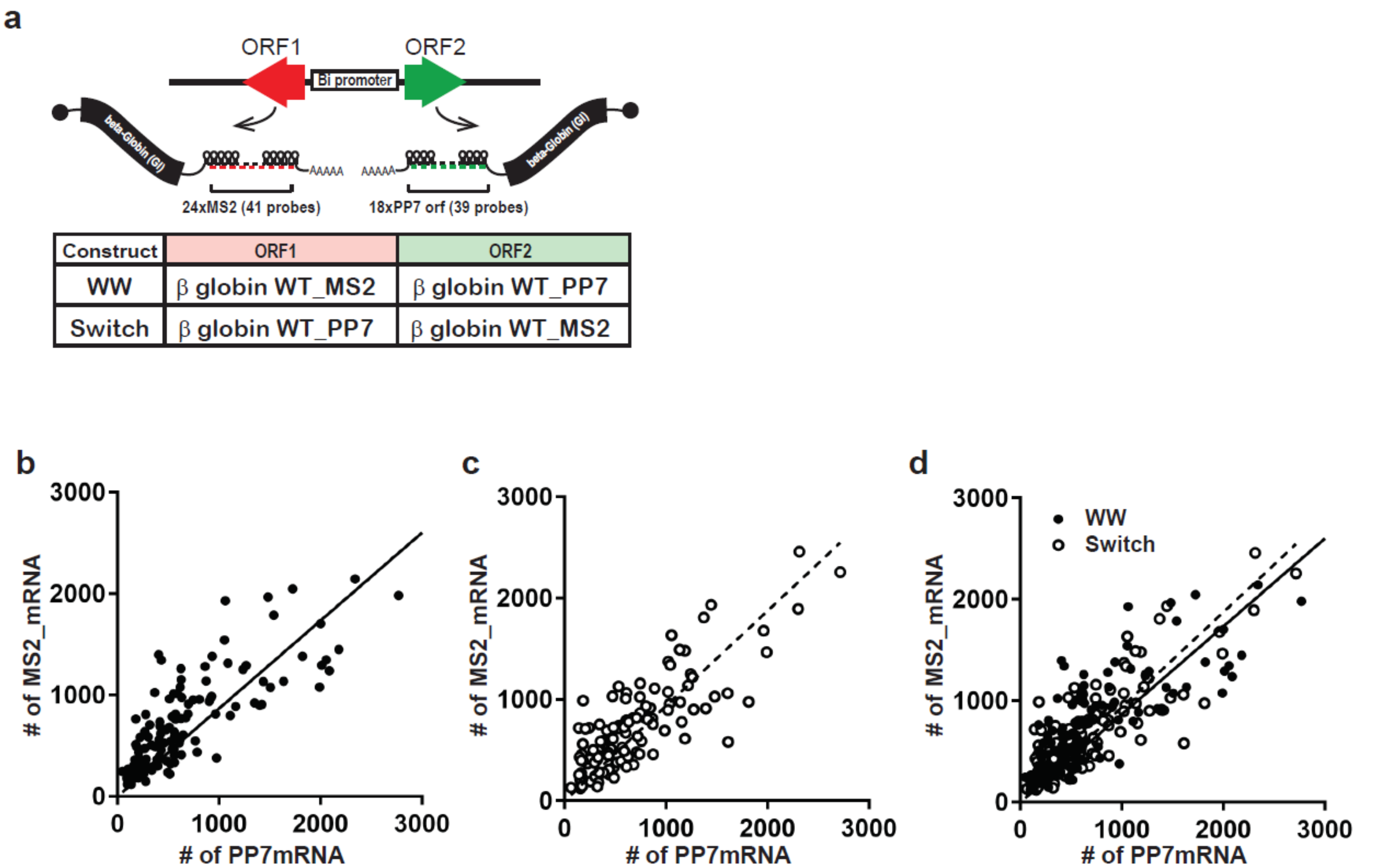

Supplementary Fig. 1 Comparable expression of β-globin mRNA with MS2 or PP7 under bidirectional promoter.

a Schematic of the smFISH to detect β-globin (Gl) mRNA expressing under PonA bidirectional promoter. These transcripts contain MS2 or PP7 sequences in the 3'UTR that hybridize with unique FISH probes. The table shows the constructs that we used in this experiment. **b**, **c**, **d** Linear correlation of Gl MS2 and Gl PP7 gene expression from WW (**b**) or Switch (**c**) construct showed that switching ORF does not affect their expressions. Lines or dotted lines indicate the regression lines of WW (**b**) or Switch (**c**) expressing cells and both datasets were superimpose in **d**. Total number of Gl MS2 and Gl PP7 mRNAs in steady-state U2OS PonA was detected using smFISH and mRNA spots were counted in cells expressing WW or Switch construct[26]. Single dots denote the number of MS2 (*y*-axis) and PP7 (*x*-axis) mRNAs from single cells. n_{cells} =114 and 111 for WW and Switch constructs. r=0.81 and 0.75 for WW and Switch constructs.

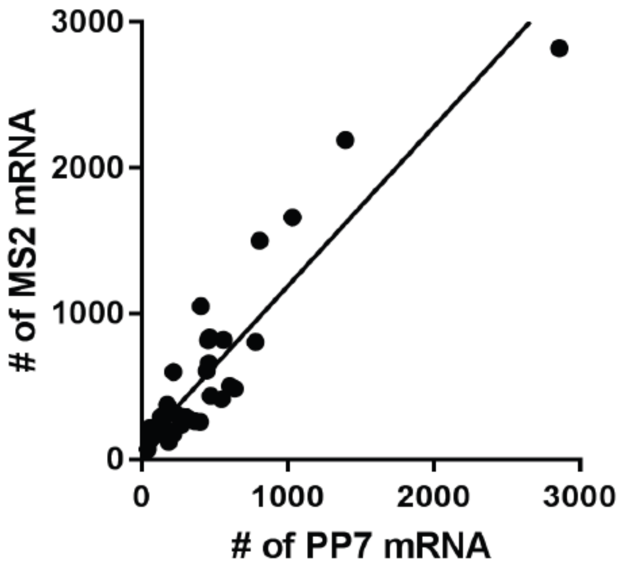

Supplementary Fig. 2 Linear correlation of Gl_MS2 and Gl_PP7 gene expression under bidirectional promoter after 1hr PonA induction. The total number of Gl_MS2 and Gl_PP7 mRNAs were detected using smFISH and single mRNA spots were counted in U2OS PonA cells expressing WW. Single dots in scatter plot denote the number of MS2 (y-axis) and PP7 (x-axis) mRNAs from single cells. n_{cells} =35. Pearson r=0.9175, R squared= 0.84. Peason r and R square were calculated by Graph Pad Prism software. Lines indicate the regression lines of WW.

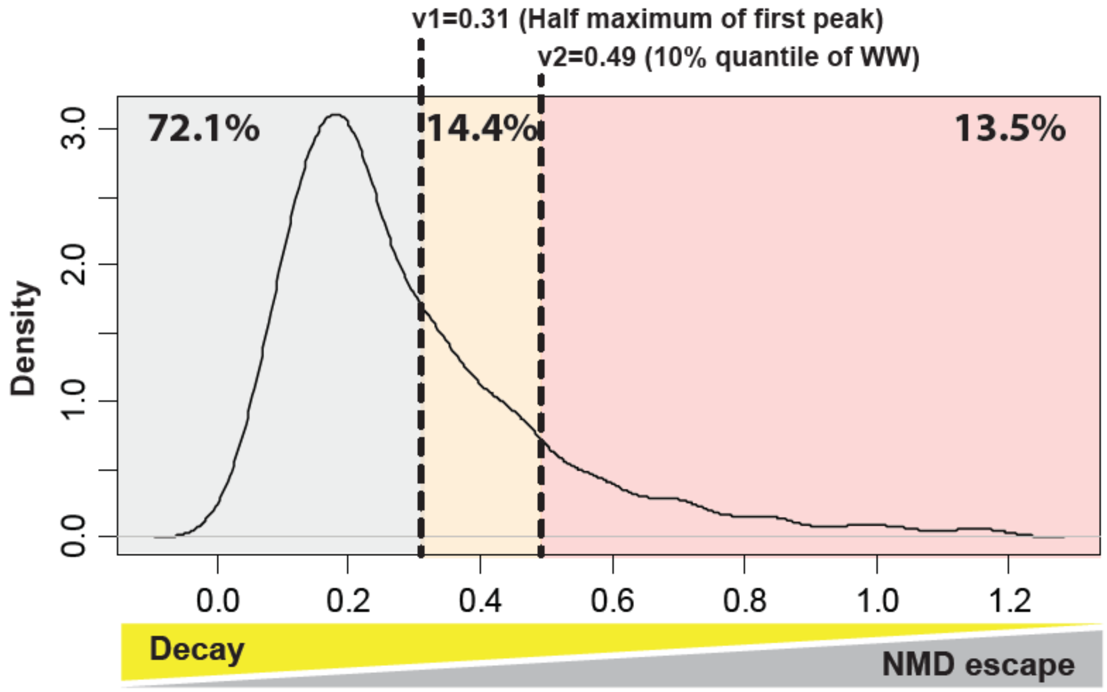

Supplementary Fig. 3 Density plot of NMD efficiency in the WP expressing cells. The level of NMD efficiency (x-axis) was calculated as the number of PTC-containing mRNA (PP7 in 3'UTR) normalized by the number of wild-type mRNA (MS2 in 3'UTR). The level of NMD escape was defined as the 10% quantile of wild-type mRNA with PP7/ wild-type mRNA with MS2 ratio (v2), and modulate escape was defined at the point of half maximum of first-peak width (v1). The analysis was performed using R (R version 4.1.0) and R studio (Version 1.4.1717).

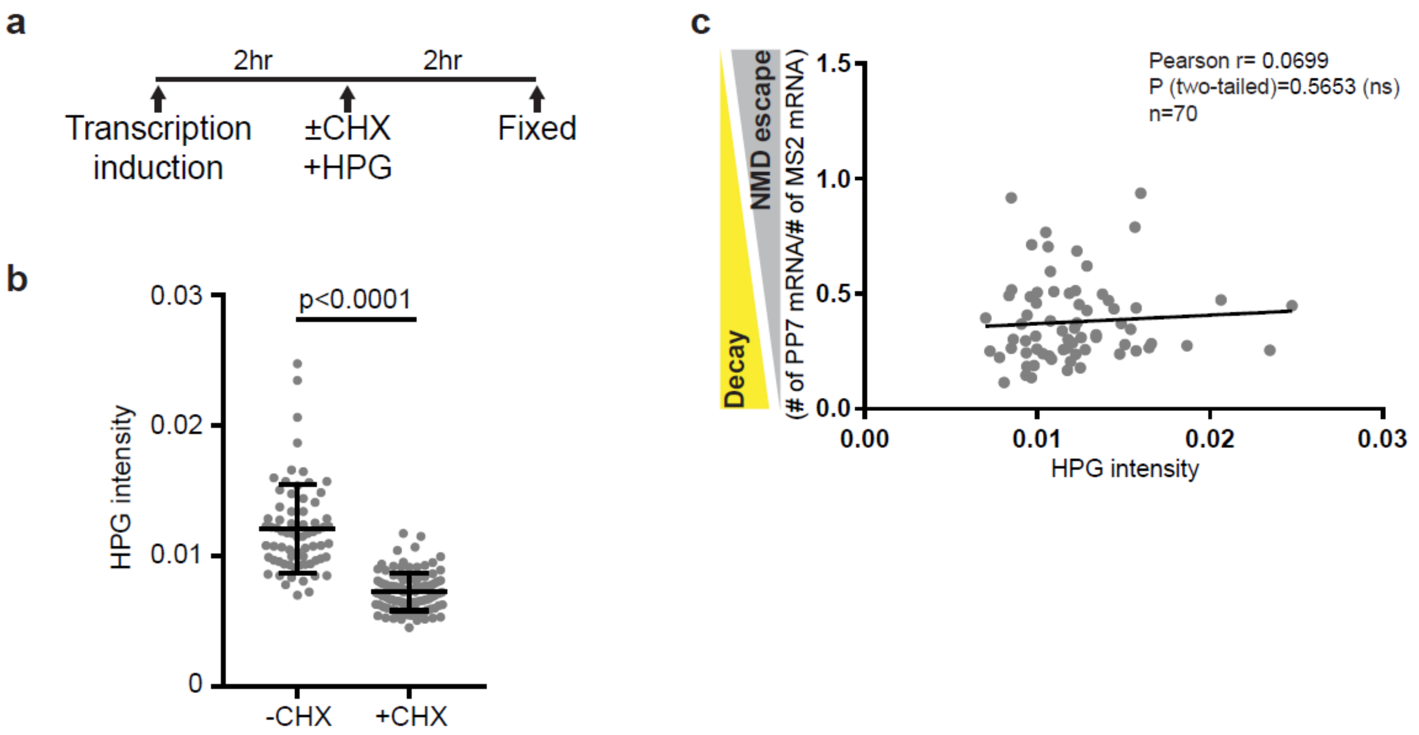

Supplementary Fig. 4 NMD efficiencies do not correlate with translation activity.

Single-cell detection to correlate translation activity and NMD. Translation activity in each cell was determined using the Click-it HPG system. **a** The timeline of transcription induction by supplement of PonA and labeling of nascent peptides with HPG incorporation. CHX, Cycloheximide; HPG, L-Homopropargylglycine. **b** Click-it HPG successfully detected translation reduction. The translation inhibition using CHX decreased HPG incorporation detected by the Click-it labeling system. n_{cells} =70 and 106 for -CHX and +CHX. P values were determined using two-tailed unpaired t-tests. Error bars = Standard deviation from cell populations. **c** No correlation was found between NMD escape and translation activity. The level of NMD escape in single cells was calculated by the number of PTC-containing Gl mRNAs normalized by the number of wild-type Gl mRNAs that were detected with smFISH. The line indicates the regression line. The correlation test between NMD efficiency and the level of HPG was performed by Graphpad prism software.

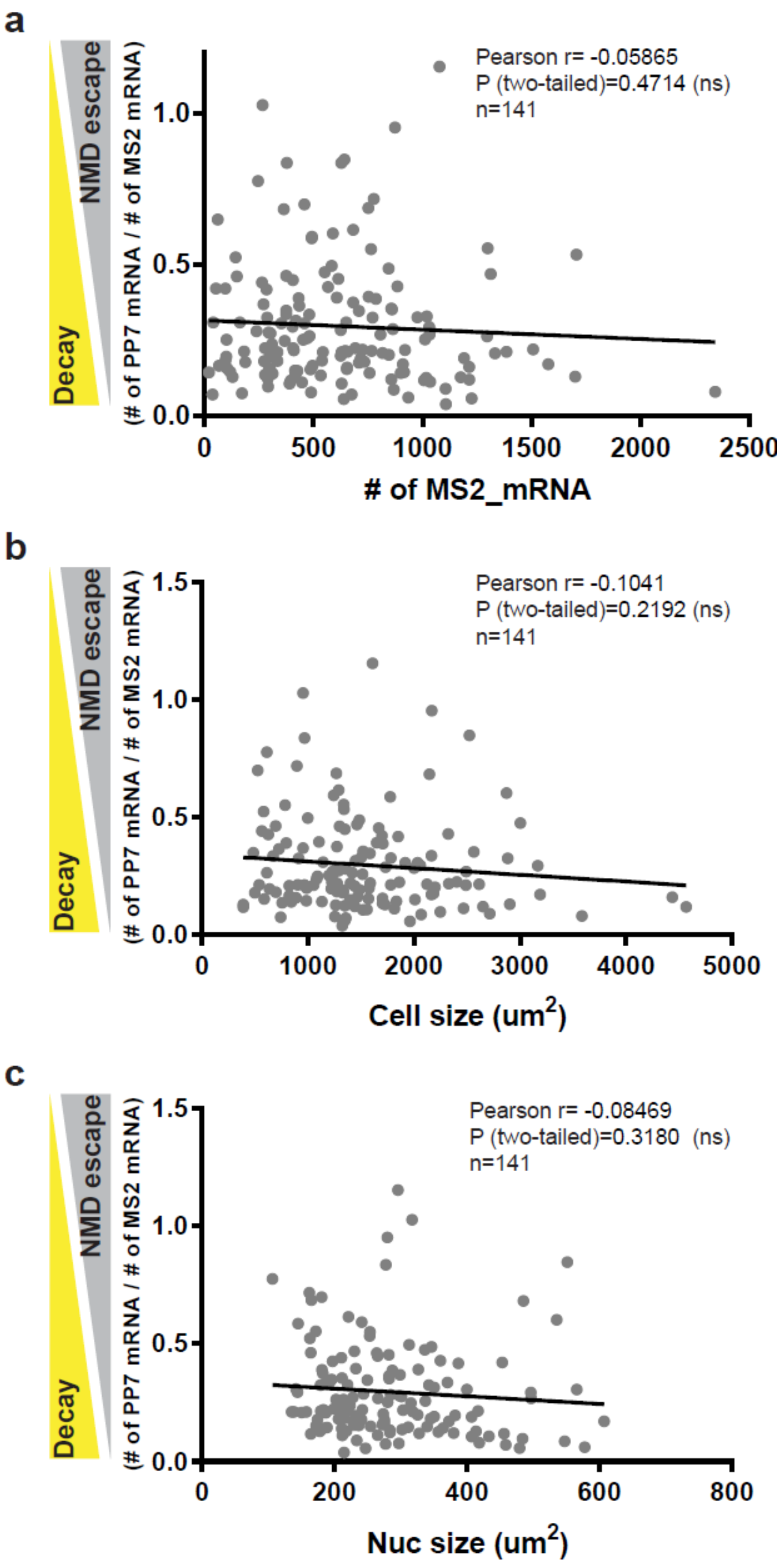

Supplementary Fig. 5 The level of NMD escape does not correlate with the level of mRNA expression, the cell size, or nuclear size.

The level of NMD escape in single cells was calculated by the number of PTC-containing Gl PP7 mRNAs normalized by the number of wild-type Gl MS2 mRNAs. **a** The level of mRNA expression was estimated by the level of Gl WT MS2 mRNA which is not degraded by NMD. **b** The cell size was measured using FISH-quant based on the cell outline area. **c** The area of the nucleus was defined using the threshold of DAPI staining. The lines indicate the regression lines. Correlation test was performed by Graphpad prism software.

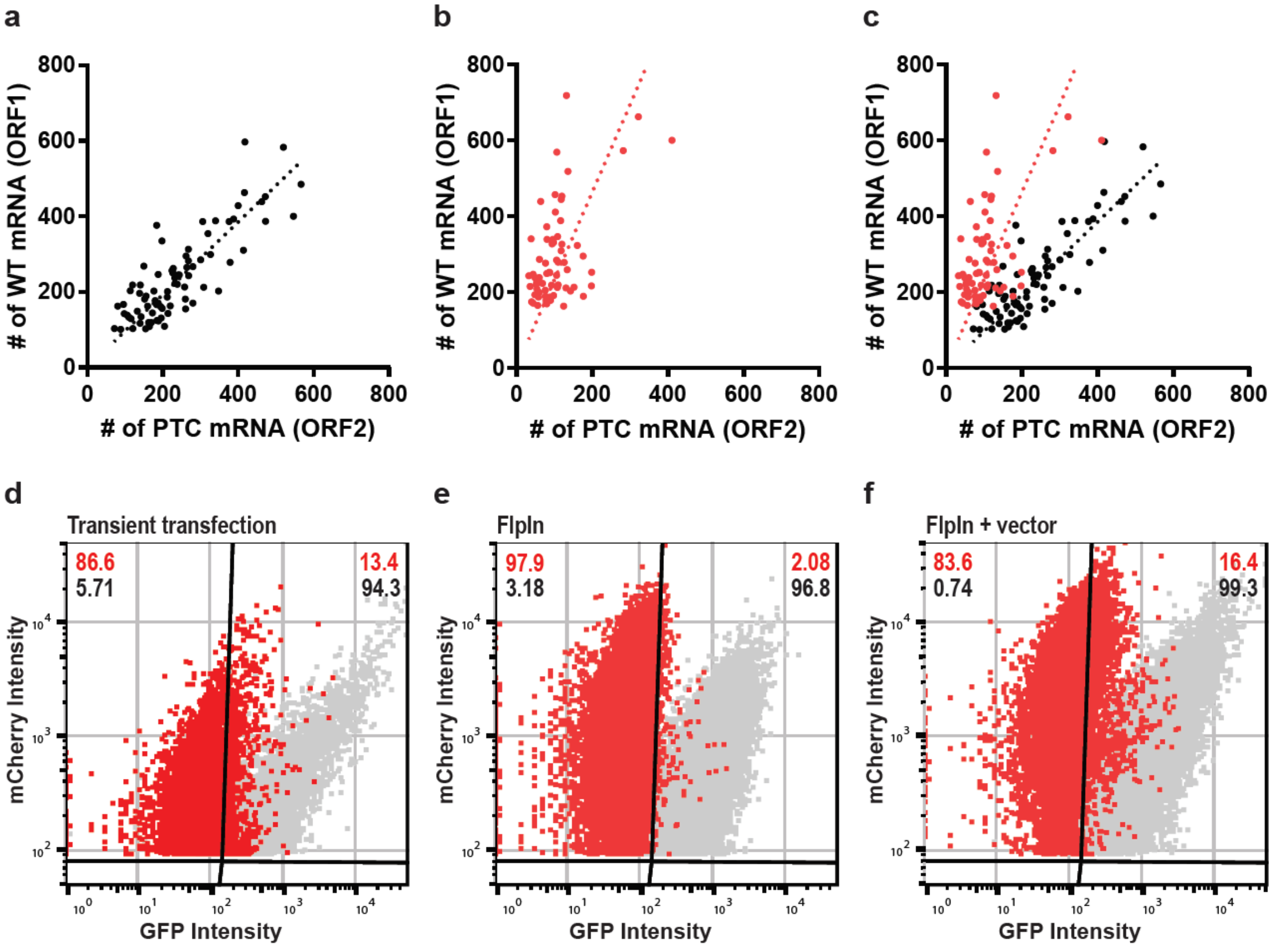

Supplementary Fig. 6. Single-cell analysis of NMD efficiency in transient transfected or Flp-In stably expressing cells

a, b, c WW or WP construct in Figure 1 was stably integrated into a single genomic location into Flp-In U2OS ponA cell line. The Scatter plots show the number of GI wild-type (ORF1) and GI wild-type or PTC (ORF2) mRNAs expressing from WW (**a**, black) or WP (**b**, red) in Flp-In U2OS ponA cells. Both were superimposed (**c**). Each dot in scatter plots denotes the number of GI wild-type (ORF1; y-axis) and wild-type or PTC (ORF2; x-axis) mRNAs in a single cell. n_{cells} =81 and 70 for WW and WP. Slope=0.96 and 2.31, R-square=0.7 and 0.31, Pearson r=0.84 and 0.56 for WW and WP. Dotted lines indicate regression lines. **c**, **d**, **e** FP construct (FP-WW; Wild-type and Wild-type or FP-WP; Wild-type and PTC) was transiently transfected (**d**) or stably integrated into a single genomic location (**e**, **f**) into U2OS ponA cell line. Non-fluorescent control plasmid, WW was transiently transfected into FP-WW or FP-WP Flp-In stably cell line (**f**) to test if the stimulation of transient transfection induces the NMD escape. Transcription was induced with 20nM ponasterone A and the intensities of mCherry and GFP were detected using BD FACSArea II. 20,000 cells were analyzed and single cells were determined using SSC and FSC, and live cells were selected as DAPI negative cells. mCherry positive cells were analyzed.

The numbers on each left or right top corner indicate the cell fractions (%) of FP-WW (black) or FP-WP (red) in the separated areas by the solid black lines.

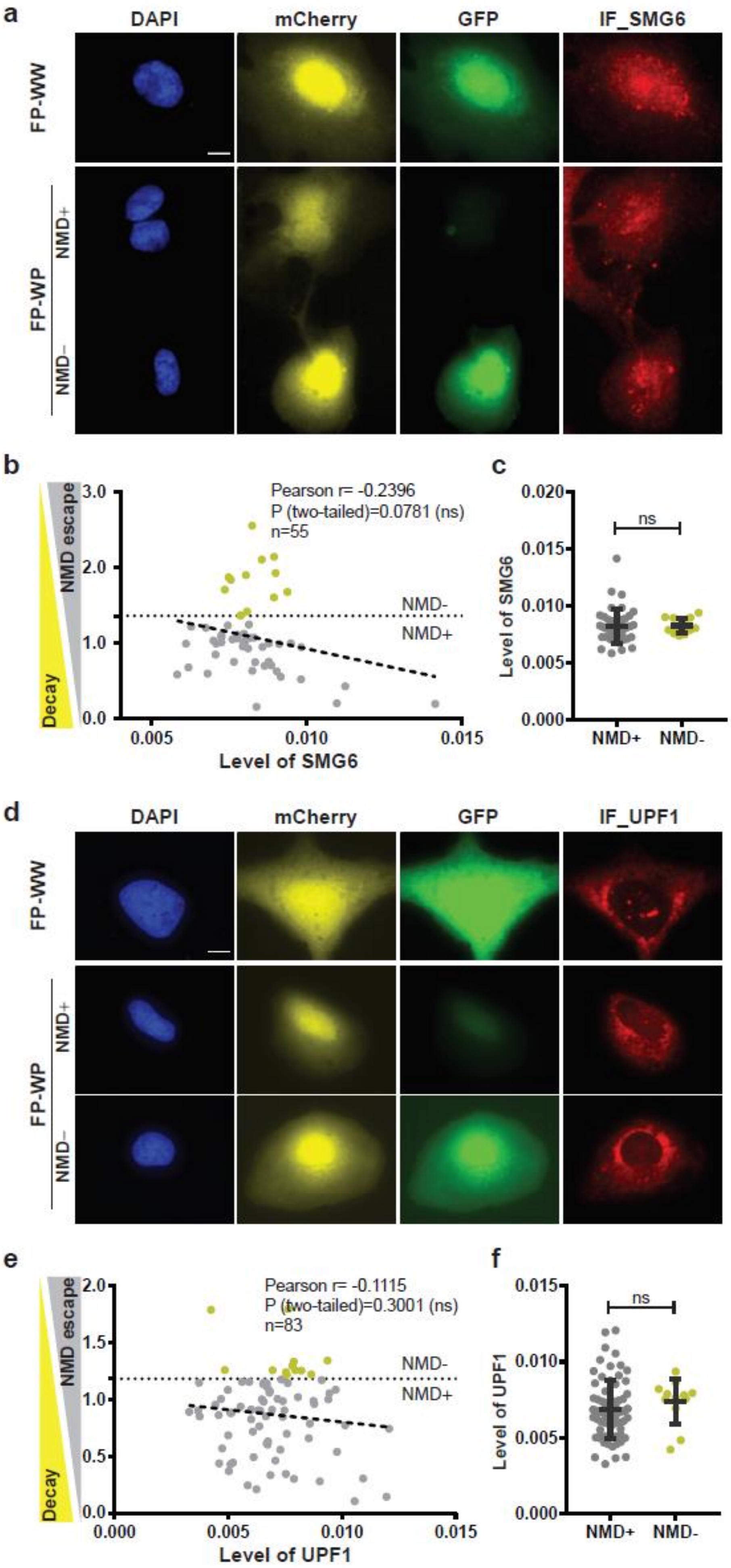
a
DAPI
mCherry
GFP
IF_SMG6
FP-WW
FP-WP
NMD+
NMD-
b
Pearson r= -0.2396
P (two-tailed)=0.0781 (ns)
n=55
NMD escape
Decay
NMD-
NMD+
Level of SMG6
c
ns
Level of SMG6
NMD+
NMD-
d
DAPI
mCherry
GFP
IF_UPF1
FP-WW
FP-WP
NMD+
NMD-
e
Pearson r= -0.1115
P (two-tailed)=0.3001 (ns)
n=83
NMD escape
Decay
NMD-
NMD+
Level of UPF1
f
ns
Level of UPF1
NMD+
NMD-

Supplementary Fig. 7 The level of NMD escape does not correlate with the protein level of SMG6 and UPF1. a Simultaneous detection of Fluorescence NMD reporter and SMG6 or **d** UPF1. FP construct (FP-WW; Wild-type and Wild-type or FP-WP; Wild-type and PTC) was transiently transfected into U2OS ponA cell line. Transcription was induced with 20nM ponasterone A for 24 hours. Bar = 10 µm. No correlation between NMD efficiency and the level of IF detected SMG6 (**b**) or UPF1 (**e**). **c** The levels of SMG6 in FP-WP expressing cells with distinct NMD efficiencies. The levels of SMG6 (**c**) or UPF1 (**f**) in FP-WP expressing cells with distinct NMD efficiencies. Cells were transfected, imaged and analyzed as described in Figure 3. The dashed lines indicate the regression lines. n_{cells} =55 (42 and 13 in NMD+ and NMD -) and 83 (71 and 12 in NMD+ and NMD -) for SMG6 and UPF1 IF. P values were determined using two-tailed unpaired t-tests (ns; not significant). Error bars = Standard deviation in cell populations.

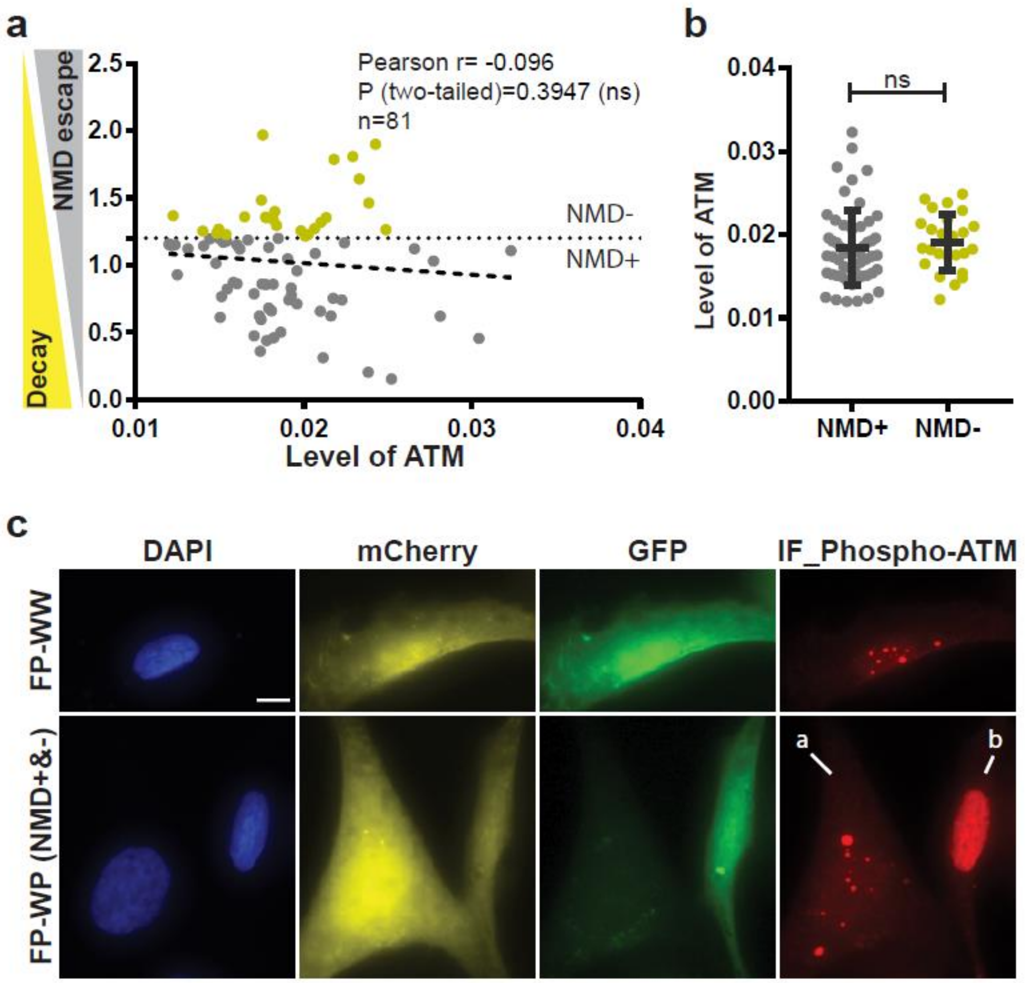

Supplementary Fig. 8 No correlation of NMD efficiency with the level of ATM.

a The level of NMD escape does not correlate with the protein level of ATM. Cells were-transfected, imaged and analyzed as described in Figure 3. The level of ATM was determined by immunofluorescence using anti-ATM antibody followed by Alexa Fluor 647 linked anti-rabbit IgG. No correlation between NMD efficiency and the level of IF detected ATM was found in FP-WW expressing cells. The dashed line indicates the regression line. **b** The level of ATM in FP-WP expressing cells with distinct NMD efficiencies. n_{cells} =81 (56 and 25 in NMD+ and NMD -). P values were determined using two-tailed unpaired t-tests (ns; not significant). Error bars = Standard deviation in cell populations. **c** Simultaneous detection of Fluorescence NMD reporter and ATM phosphorylation at S1981. Cells were transfected, imaged and analyzed as described in Figure 3. Phospho-ATM S1981P was detected by immunofluorescence using anti-ATM S1981P antibody followed by Alexa Fluor 647 linked anti-mouse IgG. Bar = 10 µm. (a) shows a cell with efficient NMD (lower EGFP expression) and (b) shows a cell with NMD escape (higher EGFP expression).

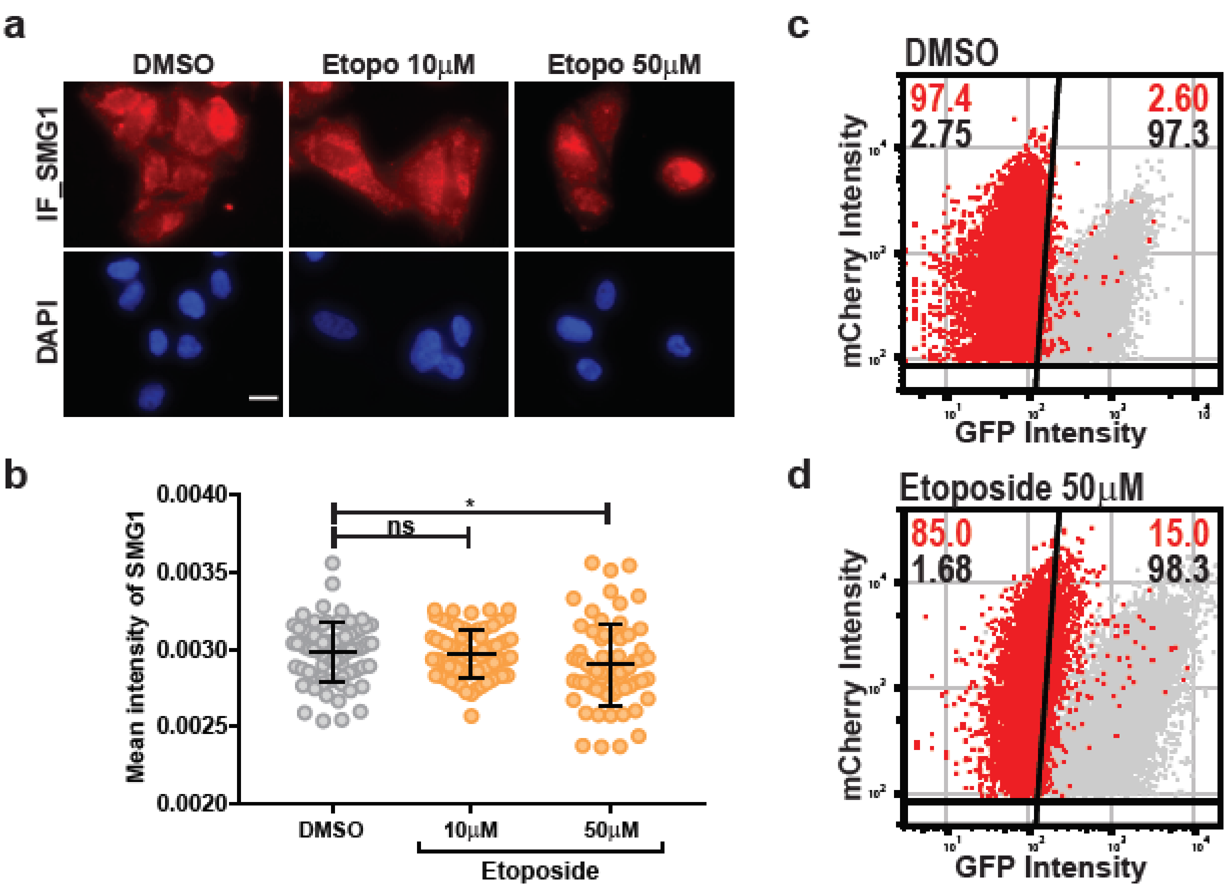

Supplementary Fig. 9 SMG1 is decreased by a DNA damage inducer, Etoposide.

a SMG1 detection in U2OS cells by IF under the treatment of etoposide (Etopo). Cells were treated with etoposide for 24h with the indicated concentrations. DMSO was used for a control. Immunodluorescence against SMG1 was performed as described in Figure 4. Bar = 10 µm. **b** The mean intensity of SMG1 in cytoplasm was detected in **a**. n_{cells} =76, 76, 66 for DMSO, 10μM, or 50μM etoposide treatment. Single dots denote fluorescence intensity of SMG1 intensity in individual cells. P values were determined using two-tailed unpaired t-tests (*P=0.0370). Error bars = Standard deviation in cell populations. The statistical analysis was performed by Graphpad prism software. **c, d** Single-cell analysis of NMD efficiency using flow cytometry with DMSO (**c**) or 50 µM Etoposide (**d**) treatment. FP-WW (expressing wild-type and wild-type, shown in gray dots) or FP-WP (expressing wild-type and PTC, shown in red dots) was stably integrated in Flp-In U2OS ponA cell line. The intensities of mCherry and GFP were detected using BD FACSArea II. 20,000 cells were analyzed and single cells were determined using SSC (side scatter) and FSC (forward scatter), and live cells were selected as DAPI negative cells. mCherry positive cells were analyzed. The numbers on each left or right top corner indicate the cell fraction (%) of FP-WW (black) or FP-WP (red) in the separated areas by the solid black lines.

Supplementary Table 1: Probe list for smFISH

MS2 Probe Sequence (5' to 3')	PP7 Probe Sequence (5' to 3')
tgattgtgaagtgtcgggtg	tacaaaattggctctcggcg
tccacccttgtgtattgtac	ggttttgtcgagaaactggc
tgtaatgtgtctggagggtg	tgtatacatagcggagggac
gcttctgtttgattggattt	tgaccttcgatgactgtctg
gatggtgattccttgttgta	catatggtctgctcccatac
gtatattgcacagggaatcc	taatagccgcgacattcttt
gatattcgggaggcgtgatc	cctctgtccgtgtacaaaaa
acgcactgaattcgaaagcc	acaggtacagacggacgagg
attcgactctgattggctgc	agtaataagcgctgagcgcg
ctcttcgcgaaagtcgactt	atatcctctgtcggcgtatc
taagaatggcgcgaaggctg	gacatttgggaacagtcgga
gtaggggagagtgtggtttg	tataaggacggaggccatac
caggaacgctgatgctgttc	agtaaaggttcttggtcgac
ttttcttgagttgggtactg	tctctgggaccagatacaag
tgatgctgcatggggacata	acgcgttcccaaatcttttg
ttggggatgtattcttgggg	ttgtatgattggcctcggag
ttggtgctcggatgtgattt	tgcgcacagagcataatact
aagaaacaacactccgagcc	ttgtacataaccggagcctc
atggagggtttgtccagttg	tcgcgggtacacaaactgat
tttgtcttgttggtgagagt	catacggtgtgctggtaagt
ctgatgctgcttcgagaaga	tatatggacagggacaggga
gtatgctcgagtgtttcgaa	ggtctgtcgggatacataac
gatcgtccacccaagaaata	ctcgacaaataccgacgacc
aattcgtgagagcatgggtg	acaattttgtacttgcgtgt
tcgtattggacgtggaacga	catatgtcctgcgagatatc
tcgtgatcccgaaaggtaag	aatagagatctgctctcgcg
atcgtgcatgcttgaatgtc	atatcctctggtgccgatag
gttgagacttgtggagcatg	aagatgctgaggcaccgagg
tgaacccatttggtagtttc	ttggacagtgacagcacatc
tttgaggtaggagtgggttc	atatgctctgctggcttgaa
ttgccagttttgtgggaaga	tgtaatgacagtggagccag
tttggtatgttggaatgggc	gattggagaagatcaggtcc
gatgctgtaccagtaattgt	atgtagtcggagggagcgag
tagtagtgagagatgtgggc	tagtctctcagcacttcgag
tgctgaacggtttggttttt	catatcgctgaccctttgat
ttgatttttccgtgtgtacc	gaacgagtagtaggggtcgc

gtctttcgtatttgtaaacc
ttgcgctggacgaaagcgtg
ccgtcggatgtttttcgtaa
ggttgtaagtttgtgggttg
ctgaggtgtttgatgtacgg

cgactctgcgttgaagagta
tttgtatctctgatggacgc

Supplementary Table 2: Primer list for RT-qPCR

	Forward	Reverse
UPF1	AGATCACGGCACAGCAGAT	TGGCAGAAGGGTTTTCCTT
UPF2	TCTCACCTGAGGACCAGTGTAC	AGCTGGAGGTGGGTTGCAGTAG
UPF3A	TACTGGAGGTGGCAAGCAGGAA	CCTGTGCTCTTTATCACTGCCG
UPF3B	GAAAGAGCCAGTGGGCAAAGTTG	CGAAGTATGCGCTCCTGATCTC
SMG1	TCGAAGTCAAGAACACGTTGA	GGGTGATGCAAAACTCACTAAA
SMG5	CACTAAGCGGCCGCTACTGAC	TCTATGCGGCCGCCTAACGTCTTGGCAACAAAGGGAC
SMG6	GATGGTCTTGCCATTCGCAGCA	TCGCTGTATCACTGGCTTGCTC
SMG7	TTTCAGGAGGCAGTGGTGGATG	CAAACTCCTCTGGAAGTGGTGTC
BAG-1	GTGAACCAGTTGTCCAAGACCTG	CAAGTGCTGACAACGGTGTTTCC
ANTXR1	ATGCCTTGTGGGTCCTACTG	GAGGTGTGGTAGGCGTTGTT
GADD45A	GGAGGAATTCTCGGCTGGAG	CGTTATCGGGGTCGACGTT
ATF4	TCAACATCGTGCGGGTGTCG	CCCGGCTTTCTTCGCAGTAG
GAS5	CTTGCCTGGACCAGCTTAAT	CAAGCCGACTCTCCATACCT
ACTB	TCCCTGGAGAAGAGCTACG	GTAGTTTCGTGGATGCCACA
GFP	TCCGCCCTGAGCAAAGAC	TTGTACAGCTCGTCCATGC
mCherry	GGCGCCTACAACGTCAAC	TTGTACAGCTCGTCCATGC
