# Supplementary material for: Cellular variability of nonsense-mediated mRNA decay
Source: Nat Commun. 2021 Dec 10;12:7203. doi: 10.1038/s41467-021-27423-0 (PMC8664836; doi:10.1038/s41467-021-27423-0)
Supplement: Supplementary file 3 — Reporting Summary [file 41467_2021_27423_MOESM3_ESM.pdf]

nature portfolio

Corresponding author(s): Robert Singer

Last updated by author(s): Oct 25, 2021

Reporting Summary

Nature Portfolio wishes to improve the reproducibility of the work that we publish. This form provides structure for consistency and transparency in reporting. For further information on Nature Portfolio policies, see our Editorial Policies and the Editorial Policy Checklist.

Statistics

For all statistical analyses, confirm that the following items are present in the figure legend, table legend, main text, or Methods section.

n/a	Confirmed	
☐	☒	The exact sample size (*n*) for each experimental group/condition, given as a discrete number and unit of measurement
☐	☒	A statement on whether measurements were taken from distinct samples or whether the same sample was measured repeatedly
☐	☒	The statistical test(s) used AND whether they are one- or two-sided<br>*Only common tests should be described solely by name; describe more complex techniques in the Methods section.*
☒	☐	A description of all covariates tested
☒	☐	A description of any assumptions or corrections, such as tests of normality and adjustment for multiple comparisons
☐	☒	A full description of the statistical parameters including central tendency (e.g. means) or other basic estimates (e.g. regression coefficient) AND variation (e.g. standard deviation) or associated estimates of uncertainty (e.g. confidence intervals)
☐	☒	For null hypothesis testing, the test statistic (e.g. *F*, *t*, *r*) with confidence intervals, effect sizes, degrees of freedom and *P* value noted<br>*Give P values as exact values whenever suitable.*
☒	☐	For Bayesian analysis, information on the choice of priors and Markov chain Monte Carlo settings
☒	☐	For hierarchical and complex designs, identification of the appropriate level for tests and full reporting of outcomes
☐	☒	Estimates of effect sizes (e.g. Cohen's *d*, Pearson's *r*), indicating how they were calculated

Our web collection on statistics for biologists contains articles on many of the points above.

Software and code

Policy information about availability of computer code

Data collection	For the images acquired at the wide-field microscope, Metamorph acquisition software 7.8.10.0 was used.<br>For the cell sorting or flow cytometry analysis, BD LSRII or BD FACSArea II was used.<br>For the western blot, Odyssey imaging system (LI-COR Biosciences) was used.
Data analysis	Flow cytometry analysis was performed using FlowJo v10.7.1.<br>The fluorescence intensities of IF were analyzed using CellProfiler3.1.9.<br>Detection of the single-molecule mRNAs using smFISH was performed by FISH-quant v3.<br>The band intensities in western blot was measured using Fiji/ImageJ 1.53c.<br>Graphpad prism version 7.04 was used for the statistical analysis.<br>R (R version 4.1.0) and R studio (Version 1.4.1717) were used to make a density plot and determined the fraction of cells in Figure S3.

For manuscripts utilizing custom algorithms or software that are central to the research but not yet described in published literature, software must be made available to editors and reviewers. We strongly encourage code deposition in a community repository (e.g. GitHub). See the Nature Portfolio guidelines for submitting code & software for further information.

Data

Policy information about availability of data

All manuscripts must include a data availability statement. This statement should provide the following information, where applicable:

- Accession codes, unique identifiers, or web links for publicly available datasets
- A description of any restrictions on data availability
- For clinical datasets or third party data, please ensure that the statement adheres to our policy

The data generated during this study are provided as a Source Data file.

Field-specific reporting

Please select the one below that is the best fit for your research. If you are not sure, read the appropriate sections before making your selection.

☒ Life sciences ☐ Behavioural & social sciences ☐ Ecological, evolutionary & environmental sciences

For a reference copy of the document with all sections, see nature.com/documents/nr-reporting-summary-flat.pdf

Life sciences study design

All studies must disclose on these points even when the disclosure is negative.

Sample size	The sample size was not statistically predetermined, and provided in the figure legends. For the imaging analysis, the sample size was determined on the sample availability. The qPCR and western blots, sample size was chosen in agreement with common practice in the field N=3.
Data exclusions	The cells that were not transfected or not expressed with the reporter gene were excluded from analyses.
Replication	Imaging and flowc ytometry assay was performed 2-3 times.<br>qPCR was performed with 3 technical replicates.
Randomization	The cells were randomly selected using the internal control (wild-type) using bi-directional promoter and analyzed.
Blinding	This study demonstrated a single-cell assay in the entire cell population from randomly selected cells using internal control.

Reporting for specific materials, systems and methods

We require information from authors about some types of materials, experimental systems and methods used in many studies. Here, indicate whether each material, system or method listed is relevant to your study. If you are not sure if a list item applies to your research, read the appropriate section before selecting a response.

Materials & experimental systems

n/a	Involved in the study
☐	☒ Antibodies
☐	☒ Eukaryotic cell lines
☒	☐ Palaeontology and archaeology
☒	☐ Animals and other organisms
☒	☐ Human research participants
☒	☐ Clinical data
☒	☐ Dual use research of concern

Methods

n/a	Involved in the study
☒	☐ ChIP-seq
☐	☒ Flow cytometry
☒	☐ MRI-based neuroimaging

Antibodies

Antibodies used	anti-UPF1 antibody (1/1000 dilution, gift from Dr. Maquat. This antibody was produced in Maquat lab), anti-phospho UPF1 antibody (1/200 dilution, Millipore, 07-1016, lot:3241225), anti-SMG1 antibody (1/200 dilution, Cell Signaling 9149S, lot:1) or anti-SMG6 antibody (1/200 dilution, invitrogen PA5-60165, lot:VJ3105234A), anti-ATM antibody (1/200 dilution, Novus Biologicals, NB100-104SS, lot:U-2), anti-ATM S1981P antibody (1/500 dilution, Active Motif #39530, lot:01008001) , anti-mCherry antibody (1/1000 dilution, Novus Biologicals, NBP2-25157SS, lot:22614), anti-GFP antibody (1/500 dilution, Roche, REF 11814460001, lot:Unknown), or anti-beta-actin antibody clone AC-15 (1/1000 dilution, Sigma, A1978), Alexa Fluor 647 secondary anti-rabbit IgG antibody (1/1000 dilution,Thermo Fisher Scientific, cat# A21245), Alexa Fluor 647 secondary anti-mouse IgG antibody (1/1000 dilution, Thermo Fisher Scientific, cat# A21236) , IRDye® 680RD Donkey anti-Mouse IgG Secondary Antibody (1/10,000 dilution, LI-COR, 926-68072), and IRDye® 800CW Goat anti-Rabbit IgG Secondary Antibody (1/10,000 dilution, LI-COR, 926-32211)

Validation	The antibodies used in this study were validated in previous publications or by the companies as described below.<br>anti-UPF1 (https://pubmed.ncbi.nlm.nih.gov/25808464/, This antibody was produced in Maquat lab)<br>anti-phospho UPF1 (https://www.emdmillipore.com/US/en/product/Anti-phospho-Upf1-Ser1127-Antibody,MM_NF-07-1016?ReferrerURL=https%3A%2F%2Fwww.google.com%2F#anchor_COA)<br>anti-SMG1(https://www.cellsignal.com/products/primary-antibodies/smg-1-d42d5-rabbit-mab/9149)<br>anti-SMG6 (https://www.thermofisher.com/antibody/product/SMG6-Antibody-Polyclonal/PA5-60165)<br>anti-ATM (https://www.novusbio.com/products/atm-antibody_nb100-104)<br>anti-ATM S1981P (https://www.activemotif.com/catalog/details/39529/atm-phospho-ser1981-antibody-mab-clone-10h11-e-12)<br>anti-mCherry (https://www.novusbio.com/products/mcherry-antibody_nbp2-25157)<br>anti-GFP (https://www.sigmaaldrich.com/US/en/product/ROCHE/11814460001?gclid=Cj0KCQjw0emHBhC1ARIsAL1QGNdizJDzzsXpHCT55tVPBk9Vxev8yjFAU5WN-1bE_ImH5GNEr_F3ATQaAnQiEALw_wcB)<br>anti-beta-actin (https://www.sigmaaldrich.com/US/en/product/sigma/a1978?gclid=Cj0KCQjw0emHBhC1ARIsAL1QGNe_vNCgKWV-Ibw_IEaNy_11gmEo21DBDKqZlTHHUSA3RbHGDN1KhrsaArIIEALw_wcB)<br>Alexa Fluor 647 secondary anti-rabbit IgG antibody (https://www.thermofisher.com/antibody/product/Goat-anti-Rabbit-IgG-H-L-Highly-Cross-Adsorbed-Secondary-Antibody-Polyclonal/A-21245)<br>Alexa Fluor 647 secondary anti-mouse IgG antibody (https://www.thermofisher.com/antibody/product/Goat-anti-Mouse-IgG-H-L-Highly-Cross-Adsorbed-Secondary-Antibody-Polyclonal/A-21236)<br>IRDye® 680RD Donkey anti-Mouse IgG Secondary Antibody (https://www.licor.com/bio/reagents/irdye-680rd-donkey-anti-mouse-igg-secondary-antibody)<br>RDye® 800CW Goat anti-Rabbit IgG Secondary Antibody (https://www.licor.com/bio/reagents/irdye-800cw-goat-anti-rabbit-igg-secondary-antibody)

Eukaryotic cell lines

Policy information about cell lines

Cell line source(s)	U2OS cells (ATCC, HTB-96™)<br>HEK 293T cell (ATCC, CRL-3216™)<br>U2OS Flp-In cell line is gift from Dr. Senecal.<br>Human U2OS PonA and Flp-In U2OS PonA cell line was generated by stable transfection of pERV3, which expresses synthetic VP16-glucocorticoid/ecdysone receptor (VgEcR) and retinoid-X-receptor (RXR). Human embryonic kidney 293T PonA cell was generated by stable transfection of pERV3-zeocin vector, which is replaced neomycin drug selection gene in pERV3 to Zeocin gene due to the neomycin resistance of HEK293T cells.
Authentication	Short Tandem Repeat profiling, every 3 years
Mycoplasma contamination	All cell lines were tested as mycoplasma negative.
Commonly misidentified lines<br>(See ICLAC register)	HEK cells (Cell line validation has been performed by the short tandem repeat profiling).

Flow Cytometry

Plots

Confirm that:

☒ The axis labels state the marker and fluorochrome used (e.g. CD4-FITC).

☒ The axis scales are clearly visible. Include numbers along axes only for bottom left plot of group (a 'group' is an analysis of identical markers).

☒ All plots are contour plots with outliers or pseudocolor plots.

☒ A numerical value for number of cells or percentage (with statistics) is provided.

Methodology

Sample preparation	Live cells were suspended into the sorting buffer (Ca2+- and Mg2+-free PBS with 2% FBS and 0.1 µg/mL Dapi)
Instrument	BD FACSArea II
Software	FlowJo v10.7.1.
Cell population abundance	More than 90% of cells identified as live and single cells. More than 50% of cells out of 20,000 cells were identified as transfected cells.
Gating strategy	We used SSC, FSC and dapi for gating of single live cell. Gating of reporter expressing cells were identified using mCherry intensity.

☐ Tick this box to confirm that a figure exemplifying the gating strategy is provided in the Supplementary Information.
